# PRECARIOUS HOUSING BEFORE, DURING, AND AFTER AN EPISODE OF LATE-LIFE HOMELESSNESS

**DOI:** 10.1093/geroni/igad104.0206

**Published:** 2023-12-21

**Authors:** Anthony Traver, Holly Dabelko-Schoeny

**Affiliations:** The Ohio State University, Columbus, Ohio, United States; The Ohio State University, Columbus, Ohio, United States

## Abstract

This qualitative study aims to understand how the living environments occupied by older adults before, during, and after an episode of homelessness inform their access to a healthy, stable, and dignified life. Indicators of accommodation, quality, and service integration are explored using the Aging in the Right Place conceptual framework. Through partnerships with a homeless shelter, a meal site, and a mental health outreach team in Columbus, OH, demographic questionnaires and semi-structured interviews were completed with 22 older adults with an episode of late-life homelessness. Nine direct service providers were also interviewed. Interviews were analyzed using the team-based flexible coding method in Nvivo 1.6.1. Results indicated that low or no income in late life forced older adults to occupy a continuum of precarious and low-quality living environments that include shared housing, doubling up, emergency shelters, institutional settings, and living on the land. AIRP indicators are discussed for each. Conflict, death of a support person, mental and behavioral challenges, unit deterioration, rental price increases, and social isolation forced OA down the housing continuum and into homelessness. Sub-optimal conditions interacted with age to exacerbate health conditions, create social isolation, and expose OA to harm. Informal and formal relationships, emergency shelter services, vouchers, and specific behaviors and attitudes were identified as critical for securing affordable and accommodating housing in which to age. Understanding the experiences of OPEH who are striving to occupy their subjective right place to age can help service providers and policymakers meet the unique needs of precariously housed OA.

